# Supplementary material for: A siRNA targets and inhibits a broad range of SARS‐CoV‐2 infections including Delta variant
Source: EMBO Mol Med. 2022 Feb 21;14(4):e15298. doi: 10.15252/emmm.202115298 (PMC8988202; doi:10.15252/emmm.202115298)
Supplement: Supplementary file 2 — Appendix [file EMMM-14-e15298-s001.docx]

**EMM2021-15298**

**Appendix**

**Title: A siRNA targets and inhibits a broad range of SARS-CoV-2 infections including Delta variant**

**Authors:** Yi-Chung Chang^1^, Chi-Fan Yang^2^, Yi-Fen Chen^1^, Chia-Chun Yang ^2^, Yuan-Lin Chou^2^, Hung-Wen Chou^1^, Tein-Yao Chang^3^, Tai-Ling Chao^4^ , Shu-Chen Hsu^3^, Si-Man Ieong^4^, Ya-Min Tsai^4^, Ping-Cheng Liu^3^,Yuan-Fan Chin^3^, Jun-Tung Fang ^4^, Han-Chieh Kao^4^, Hsuan-Ying Lu^3^, Jia-Yu Chang^3^, Ren-Shiuan Weng^1^, Qian-Wen Tu^1^, Fang-Yu Chang^1^, Kuo-Yen Huang^5^,Tong-Young Lee^2^, Sui-Yuan Chang ^4,6^*, Pan-Chyr Yang^7,8,9^*

Correspondence to: pcyang@ntu.edu.tw

**Appendix Fig. S1 to S7**

**Appendix Table S1 to S6**

**
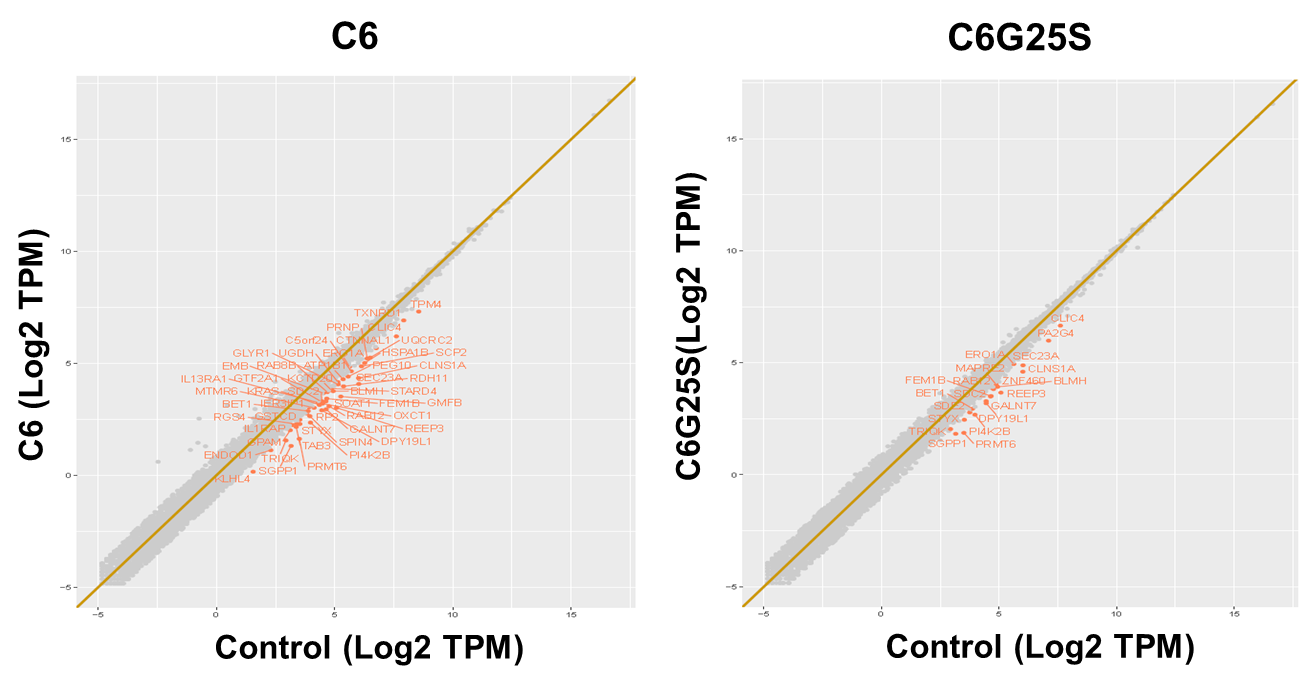
**

**Appendix Fig. S1:
Modification of C6 significantly reduced off-targets analyzed by RNA-seq.** Scatterplots display global gene expression change in C6- and C6G25S-treated BEAS-2B cells compared with no siRNA control, respectively. Red dots indicate genes down-regulated with fold change ≥2. *TPM: Transcripts per Million.


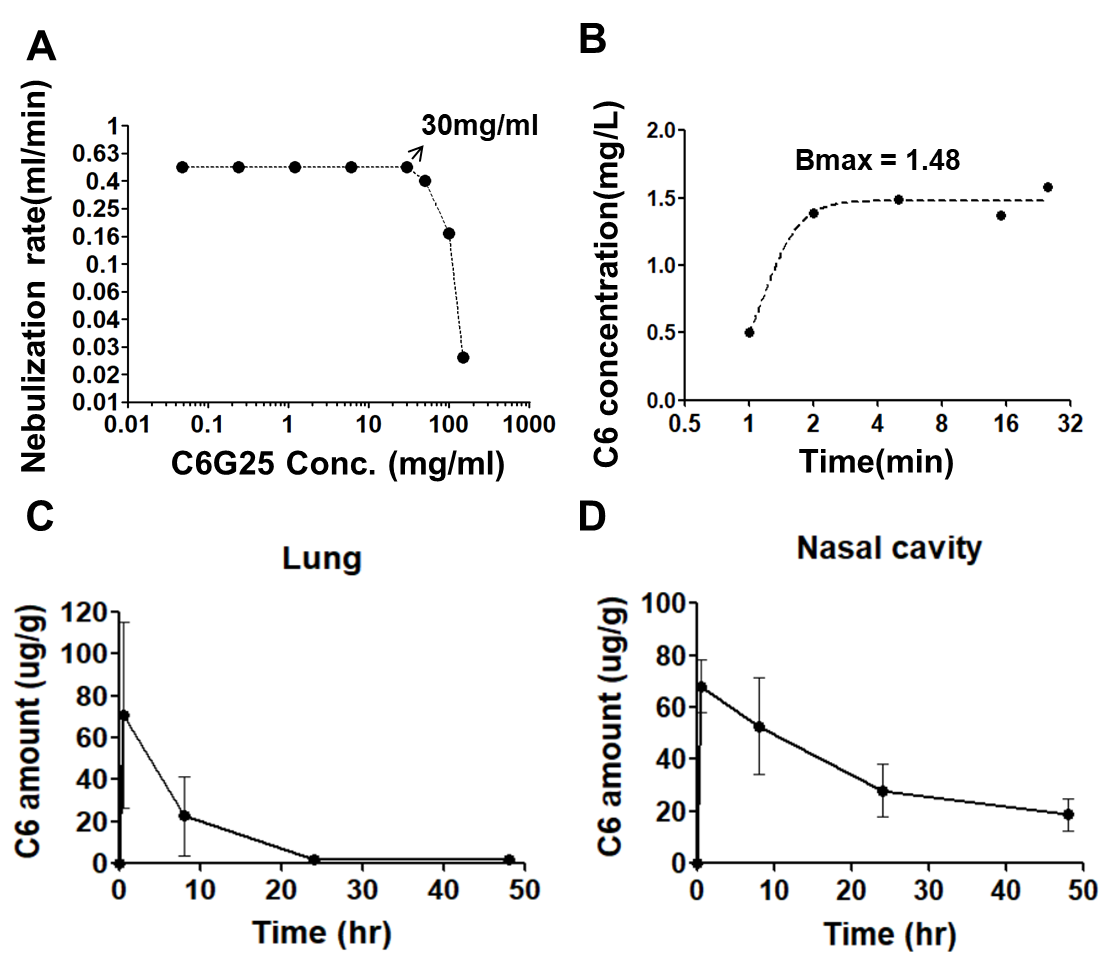


**Appendix Fig. S2:****Quantification of C6G25S in inhalation aerosol, lungs, and nasal cavities.**

A. Different concentrations of C6G25S solutions, including 150, 50, 40, 30, 6, 1.2, 0.24 and 0.048 mg/mL in normal saline, were analyzed for nebulization rate in a mesh nebulizer.

B. After aerosol was generated from 6 mg/mL of C6G25S in normal saline, aerosol samples were collected from the inhalation chamber using 0.5 mL syringes and passed through 100 μL nuclease-free water. C6G25S level in the nuclease-free water was subsequently determined by OD260. B_max_ represents the maximum C6G25S level.

**C, D.** C57/B6 mice (n=3 per group) administrated with 1.48 mg/L of C6G25S via AI for 30 min and followed by 50 ug C6G25S via IN. C6G25S level in lungs (B) and nasal cavities (C) was quantified at 0.5, 8, 24, and 48 hr post-delivery via both aerosol inhalation and intranasal instillation. Quantification data represent mean ± SD.


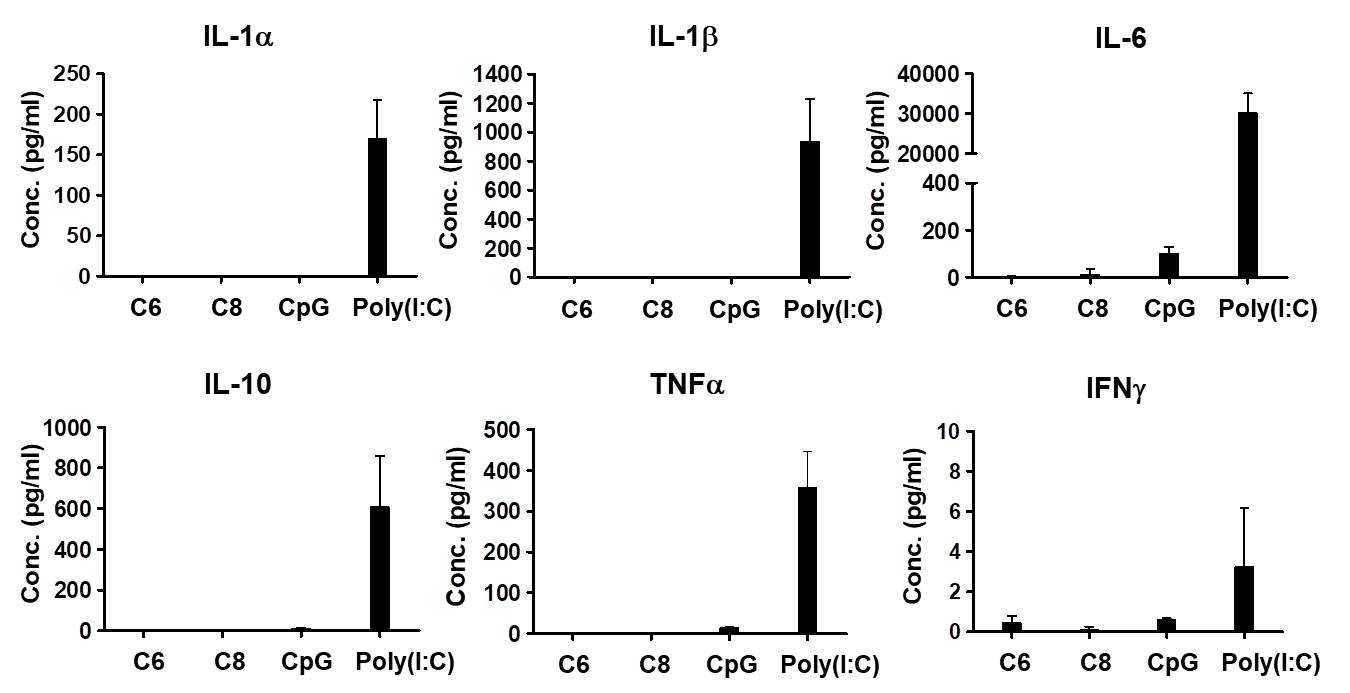


**Appendix Fig. S3:
No stimulation of inflammatory cytokines in peripheral blood mononuclear cells (PBMCs) after cocultured with 10 μM of modified C6 (C6G25S) and C8 (C8G25S) siRNA**. Cytokines IL-1α, IL-1β, IL-6, IL-10, TNF-α, and IFN-γ in the coculture medium were detected via flow cytometry analysis using Cytometric Bead Assay (CBA) Flex Set (BD Biosciences) at 40 h after treatment. CpG(1 μM) and poly(I:C) (100 μg/mL) were utilized as positive controls. Data are presented as the means ± SD of three independent experiments using PBMCs from three healthy donors. Conc. = concentration.


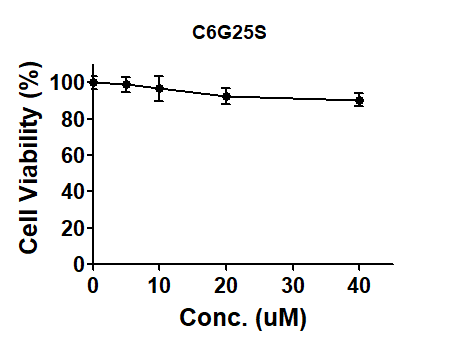


**Appendix Fig. S4:
The effect of C6G25S on the cell viability of BEAS-2B cells measured by CCK-8 assay**. Beas-2B cells were treated with various concentrations of C6G25S (40, 20, 10, 5, and 0 μM) for 24 h and cell viability was measured by CCK-8 assay according to the manufacturer’s instructions.


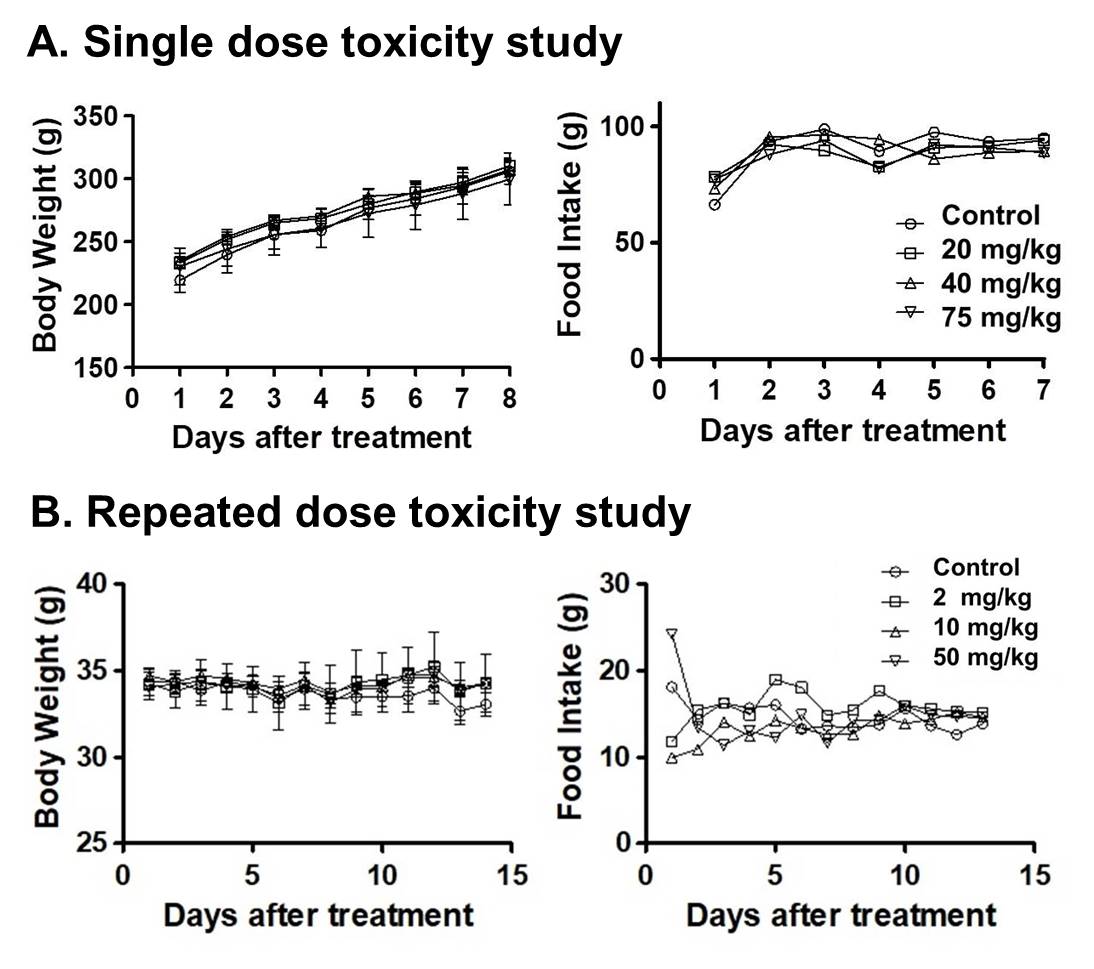


**Appendix Fig. S5:
No significant change in body weight and food intake observed during toxicology studies.**

**A.** Single-dose toxicology study. A single dose of C6G25S (0, 20, 40, and 75 mg/kg) was administered intranasally to Sprague Dawley rats at day 0 (n = 3 per group). Body weight and food intake were monitored daily for 7 days.

**B.** Repeated-dose toxicology study. ICR mice (n = 3 per group) were administered daily with the indicated concentration of C6G25S by intranasal instillation. The body weight and food intake were continuously monitored for 14 days.


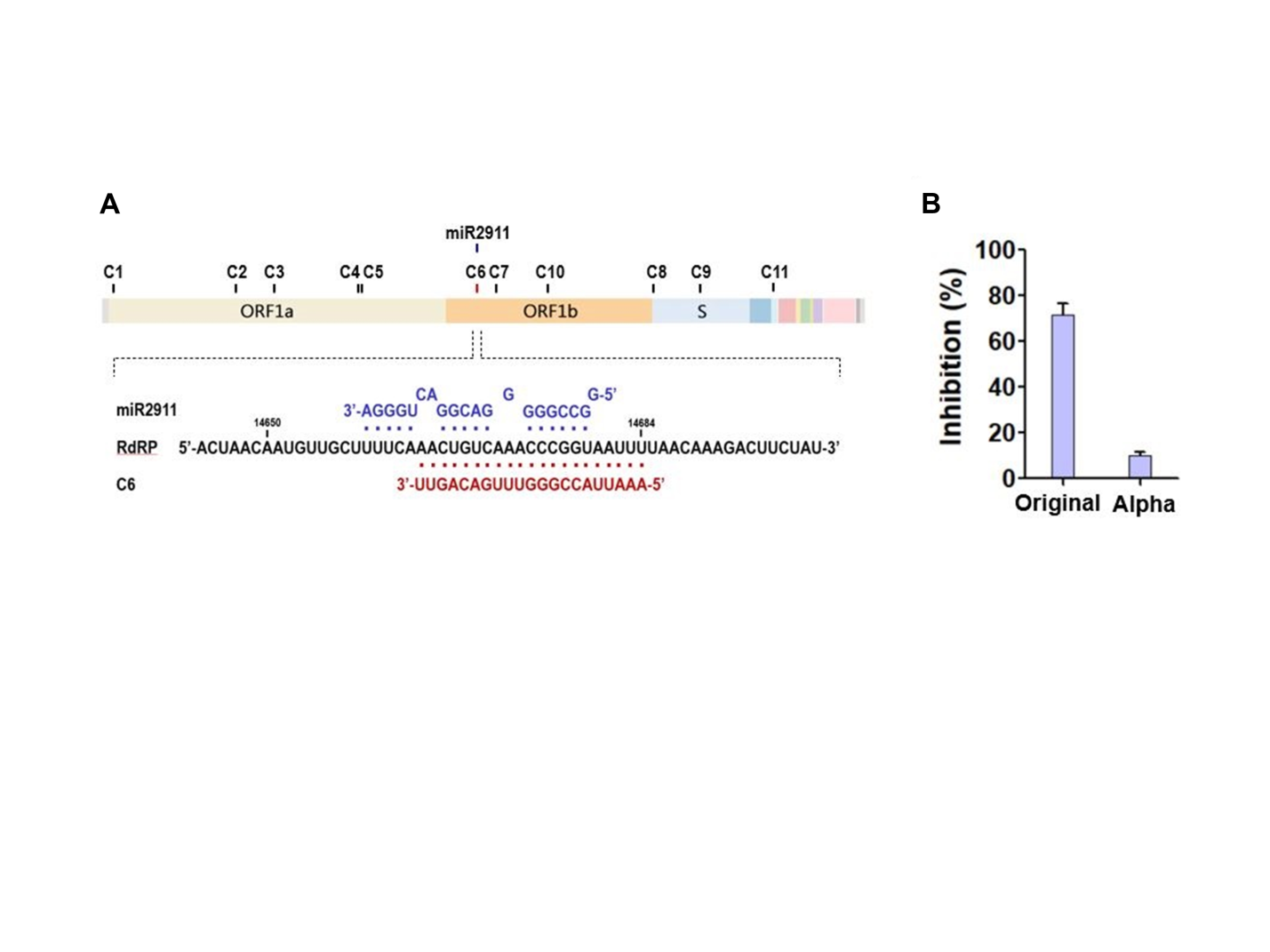


**Appendix Fig. S6:**

**miR2911 with one of the binding sites overlapping with that of C6 reduced viral RNA of original virus, but not Alpha variant.**

**A.** The locations targeted by all 11 siRNA candidates within the SARS-CoV-2 genome (accession number: NC_045512.2). The overlapping target sites of C6 and miR2911 on *RdRp* are depicted with the sequences of C6 antisense and miR2911 outlined in red and blue, respectively.

**B.** Inhibition of viral RNA caused by miR2911. Vero E6 cells were transfected with 100 nM of miR2911 and then infected with original virus and Alpha variant at a MOI of 0.1, respectively. Viral RNA was detected by RT-qPCR.


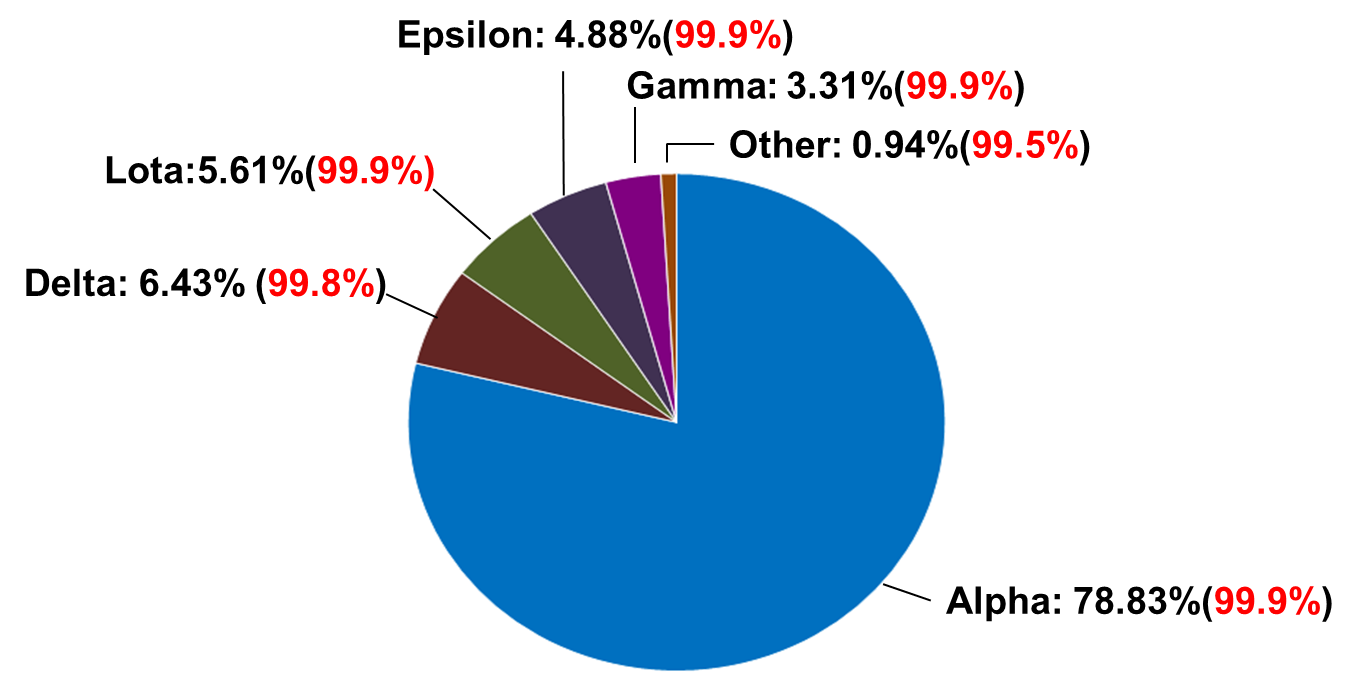


**Appendix Fig. S7:
Proportion of various virus strains and coverage rate of C6G25S**. 200,000 genome sequences of SARS-CoV2 were downloaded from NCBI virus SARS-CoV-2 Data Hub at Aug 22, 2021. The proportion of top 5 variant strains was calculated and shown in the pie chart. The percentage in red indicated the coverage rate of C6G25S for each strain and the overall coverage rate was 99.8%. The C to U transversion of Alpha variants binding to the 9^th^ nucleotide of antisense C6G25S was tolerated for the calculation of coverage rate.

**Appendix Table S1:
Histopathology of nasal cavity and lung in single-dose toxicology study.** Rats (n = 3) treated with single dose of vehicle control (D5W) or C6G25S by intranasal instillation and the blood cells were collected at day 7 after treatment. Pathological change in nose and lung were evaluated. 1 = minimal (< 10 %), 2 = mild (10–39 %), 3 = moderate (40–79 %), 4 = marked (80–100 %). No abnormal findings are labeled as “–”.

| Animal No. | Control | | | 20 mg/kg | | | 40 mg/kg | | | 75 mg/kg | | |
| --- | --- | --- | --- | --- | --- | --- | --- | --- | --- | --- | --- | --- |
| NOSE | | | | | | | | | | | | |
| Inflammation, diffuse | 2 | 2 | 3 | 1 | 2 | - | 3 | 3 | 2 | 2 | 3 | 1 |
| Degeneration, epithelium, focal | 2 | 1 | 2 | 1 | 1 | 1 | 2 | 3 | 2 | 1 | 2 | 1 |
| Necrosis, epithelium, multifocal | 1 | 1 | 2 | 2 | 1 | - | 3 | 3 | 2 | 2 | 3 | 1 |
| LUNG | | | | | | | | | | | | |
| Infiltration, mononuclear cell, focal | - | - | 1 | - | - | - | - | 1 | 1 | 1 | 1 | - |
| Inflammation, granulomatous, focal | - | - | - | - | 2 | - | - | - | - | - | - | 1 |
| Infiltration, histocyte, multifocal | - | - | - | - | - | - | 2 | - | - | - | - | - |
| The severity grading scheme: 1= minimal (<10%) 2 = mild (10-39%) 3 = moderate (40-79%) 4 = marked (80-100 %) | | | | | | | | | | | | |

**Appendix Table S2:
Blood cell analysis of a single-dose toxicity study.** Rats (n = 3) treated with single dose of C6G25S by intranasal instillation and the blood cells were collected at day 7 after treatment. Control group (vehicle alone D5W) were labeled as 1–3, 20 mg/kg-treated group as 4–6, 40 mg/kg-treated group as 7–9, and 75 mg/kg-treated group as 10–12. RBC, RBC counts with millions per microliter; HGB, hemoglobin; HCT, hematocrit; MCV, mean corpuscular volume; MCHC, mean corpuscular hemoglobin concentration; RDW-SD, RBC distribution width standard deviation; RDW-CV, RBC distribution width with coefficient of variation; RET, reticulocyte equivalent; PLT, platelet count; PDW, platelet distribution width; WBC, white blood cells; NEUT, neutrophils; LYMPH, lymphocyte; MONO, monocyte; EO, eosinophils; BASO, basophils.

| **ID** | **RBC**  **(M/uL)** | **HGB (g/dL)** | **HCT**  **(%)** | **MCV (fL)** | **MCH**  **(pg)** | **MCHC**  **(g/dL)** | **RDW-SD (fL)** | **RDW-CV (%)** | **RET (%)** | **PLT (K/uL)** | **PDW (fL)** | **WBC (k/uL)** | **NEUT (%)** | **LYMPH (%)** | **MONO (%)** | **EO**  **(%)** | **BASO (%)** |
| --- | --- | --- | --- | --- | --- | --- | --- | --- | --- | --- | --- | --- | --- | --- | --- | --- | --- |
| **#1** | 6.8 | 13.5 | 41.9 | 61.6 | 19.9 | 32.2 | 30.4 | 15.5 | 8.98 | 1033 | 8.7 | 3.96 | 10.5 | 88.1 | 0.8 | 0.3 | 0.3 |
| **#2** | 7.36 | 14.0 | 44.6 | 60.6 | 19.0 | 31.4 | 32.2 | 17.5 | 8.1 | 1064 | 8.9 | 4.52 | 26.1 | 70.6 | 2.7 | 0.4 | 0.2 |
| **#3** | 6.05 | 11.9 | 36.2 | 59.8 | 19.7 | 32.9 | 28.7 | 14.6 | 7.59 | 1061 | 8.0 | 7.08 | 18.0 | 79.0 | 2.3 | 0.7 | 0.0 |
| **#4** | 7.13 | 13.4 | 41.9 | 58.8 | 18.8 | 32.0 | 28.5 | 15.2 | 5.89 | 1064 | 8.2 | 2.56 | 14.8 | 80.5 | 2.7 | 1.2 | 0.8 |
| **#5** | 6.57 | 13.2 | 41.9 | 63.8 | 20.1 | 31.5 | 31.1 | 14.7 | 7.07 | 1021 | 9.3 | 6.83 | 12.6 | 82.3 | 3.2 | 1.6 | 0.3 |
| **#6** | 5.65 | 10.8 | 34.7 | 61.4 | 19.1 | 31.1 | 28.8 | 14.1 | 6.24 | 1055 | 8.7 | 3.76 | 13.3 | 82.7 | 2.9 | 1.1 | 0.0 |
| **#7** | 6.82 | 13.2 | 41.0 | 60.1 | 19.4 | 32.2 | 29.9 | 14.9 | 6.89 | 1086 | 9.0 | 5.44 | 22.2 | 73.2 | 3.7 | 0.9 | 0.0 |
| **#8** | 6.65 | 13.3 | 41.7 | 62.7 | 20.0 | 31.9 | 30.4 | 14.7 | 7.04 | 955 | 8.3 | 5.26 | 21.9 | 74.3 | 2.5 | 1.3 | 0.0 |
| **#9** | 6.47 | 12.8 | 40.3 | 62.3 | 19.8 | 31.8 | 30.2 | 14.7 | 6.14 | 1052 | 8.4 | 5.01 | 15.2 | 81.6 | 2.0 | 1.0 | 0.2 |
| **#10** | 6.34 | 12.6 | 40.2 | 63.4 | 19.9 | 31.3 | 31.6 | 14.9 | 6.89 | 1028 | 8.6 | 6.65 | 14.5 | 84.1 | 0.8 | 0.6 | 0.0 |
| **#11** | 6.64 | 13.0 | 42.3 | 63.7 | 19.6 | 30.7 | 30.8 | 14.3 | 6.39 | 1142 | 8.8 | 6.13 | 14.6 | 83.0 | 2.1 | 0.3 | 0.0 |
| **#12** | 6.78 | 13.2 | 42.1 | 62.1 | 19.5 | 31.4 | 30.7 | 15.3 | 6.05 | 1062 | 8.6 | 6.37 | 10.6 | 87.6 | 1.3 | 0.5 | 0.0 |

**Appendix Table S3:
Serum analysis of a single-dose toxicity study.** Rats (n = 3) treated with single dose of C6G25S by intranasal instillation and serums were collected at day 7 after treatment. Control group (vehicle alone D5W) were labeled as 1–3, 20 mg/kg-treated group as 4–6, 40 mg/kg-treated group as 7–9, and 75 mg/kg-treated group as 10–12. AST, aspartate aminotransferase; ALT, alanine aminotransferase; BUN, blood urea nitrogen; CREA, creatinine.

| **Treatment** | **ID** | **AST**  **(U/L)** | **ALT**  **(U/L)** | **BUN**  **(mg/dL)** | **CREA**  **(mg/dL)** |
| --- | --- | --- | --- | --- | --- |
| **Control** | **A2** | **45** | **36** | **12.9** | **0.23** |
|  | **A3** | **57** | **58** | **15.6** | **0.33** |
|  | **A11** | **57** | **40** | **11.6** | **0.26** |
| **2 mg/kg** | **B1** | **62** | **40** | **15.1** | **0.27** |
|  | **B9** | **49** | **46** | **15.1** | **0.29** |
|  | **B12** | **47** | **47** | **19.2** | **0.26** |
| **10 mg/kg** | **C4** | **55** | **51** | **14.5** | **0.24** |
|  | **C8** | **52** | **31** | **11.6** | **0.19** |
|  | **C14** | **46** | **39** | **11.1** | **0.24** |
| **50 mg/kg** | **D16** | **46** | **34** | **13.5** | **0.23** |
|  | **D21** | **51** | **47** | **13.8** | **0.3** |
|  | **D25** | **57** | **40** | **13.1** | **0.24** |

**Appendix Table S4:
Histopathology of major tissue in multiple-dose toxicology study.** Mice (n = 3) were intranasal administrated with 2, 10 or 50 mg/kg of C6G25S once daily for 14 days and scarified for histopathology study. The vehicle control group (D5W) were labeled as A2, A3, A11, 2 mg/kg-treated group as B1, B9, B12, 10 mg/kg-treated group as C4, C8, C14, and 50 mg/kg-treated group as D16, D21, D25. The severity grading scheme: 1 = minimal (< 10 %), 2 = mild (10–39 %), 3 = moderate (40–79 %), 4 = marked (80–100 %). No abnormal findings are labeled as “–”.

|  | **Control** | | | **2 mg/kg**  **C6G25S** | | | **10 mg/kg C6G25S** | | | **50 mg/kg C6G25S** | | |
| --- | --- | --- | --- | --- | --- | --- | --- | --- | --- | --- | --- | --- |
| **Animal No.** | **A-2** | **A-3** | **A-11** | **B-1** | **B-9** | **B-12** | **C-4** | **C-8** | **C-14** | **D-16** | **D-21** | **D-25** |
| **Heart** | **-** | **-** |  | **-** | **-** | **-** | **-** | **-** | **-** | **-** | **-** | **-** |
| **Liver** | | | | | | | | | | | | |
| Infiltration, mononuclear cell, focal | **-** | **1** | **-** | **-** | **1** | **-** | **-** | **-** | **-** | **1** | **-** | **1** |
| Necrosis, focal | **-** | **-** | **-** | **-** | **-** | **-** | **-** | **-** | **-** | **1** | **-** | **-** |
| **Spleen** | **-** | **-** |  | **-** | **-** | **-** | **-** | **-** | **-** | **-** | **-** | **-** |
| **Kidneys** | | | | | | | | | | | | |
| Infiltration, mononuclear cell, focal |  | **1** |  | **2** | **1** |  |  | **2** |  |  |  |  |
| Basophilia, tubule, cortex, focal |  |  | **1** | **1** | **1** |  |  |  |  | **1** | **1** |  |
| Cyst, cortex, focal |  |  |  |  |  |  |  |  |  |  |  |  |
| **Lung** | | | | | | | | | | | | |
| Infiltration, mononuclear cell, focal | **1** |  |  |  |  |  |  | **1** |  |  |  |  |
| Inflammation, granulomatous, focal | **2** |  |  |  |  | **1** |  |  |  |  |  |  |
| Infiltration, histocyte, multifocal |  |  |  |  |  |  |  |  |  |  |  | **1** |
| **Nasal cavity** | | | | | | | | | | | | |
| Inflammation, focal |  |  | **2** |  | **1** |  | **1** |  | **1** |  | **1** | **1** |
| Degeneration, epithelium, focal | **2** |  | **2** | **3** |  | **2** | **2** | **1** | **1** | **1** | **2** | **-** |

**Appendix Table S5:.**

**Blood cell analysis of a multiple-dose toxicity study.**

Mice (n=3) were intranasal administrated with 2, 10 or 50 mg/kg of C6G25S once daily for 14 days and blood was collected for blood cell analysis. Vehicle control group were labeled as A2, A3, A11, 2 mg/kg-treated group as B1, B9, B12, 10 mg/kg-treated group as C4, C8, C14, and 50 mg/kg-treated group as D16, D21, D25. RBC, RBC counts with millions per microliter; HGB, hemoglobin; HCT, hematocrit; MCV, mean corpuscular volume; MCHC, mean corpuscular hemoglobin concentration; RDW-SD, RBC distribution width standard deviation; RDW-CV, RBC distribution width with coefficient of variation; RET, reticulocyte equivalent; PLT, platelet count; PDW, platelet distribution width; WBC, white blood cells; NEUT, neutrophils; LYMPH, lymphocyte; MONO, monocyte; EO, eosinophils; BASO, basophils.

| **ID** | **RBC**  **(M/uL)** | **HGB (g/dL)** | **HCT**  **(%)** | **MCV (fL)** | **MCH**  **(pg)** | **MCHC**  **(g/dL)** | **RDW-SD (fL)** | **RDW-CV (%)** | **RET (%)** | **PLT (K/uL)** | **PDW (fL)** | **WBC (k/uL)** | **NEUT (%)** | **LYMPH (%)** | **MONO (%)** | **EO**  **(%)** | **BASO (%)** |
| --- | --- | --- | --- | --- | --- | --- | --- | --- | --- | --- | --- | --- | --- | --- | --- | --- | --- |
| **A2** | 8.51 | 13.0 | 42.6 | 50.1 | 15.3 | 30.5 | 27.9 | 21.3 | 3.28 | 305 | 7.1 | 7.37 | 16.9 | 79.2 | 1.6 | 2.2 | 0.1 |
| **A3** | 8.38 | 13.0 | 44.5 | 53.1 | 15.5 | 29.2 | 27.9 | 20.3 | 3.51 | 735 | 7.7 | 6.49 | 9.3 | 87.5 | 1.2 | 1.8 | 0.2 |
| **A11** | 7.99 | 12.2 | 41.4 | 51.8 | 15.3 | 29.5 | 26.1 | 19.6 | 3.36 | 813 | 7.6 | 4.57 | 22.5 | 75.3 | 0.9 | 1.3 | 0.0 |
| **B1** | 8.30 | 13.2 | 42.8 | 51.6 | 15.9 | 30.8 | 26.6 | 20.5 | 3.57 | 1254 | 6.5 | 6.03 | 13.6 | 83.7 | 1.3 | 1.2 | 0.2 |
| **B9** | 7.67 | 11.6 | 38.7 | 50.5 | 15.1 | 30.0 | 26.2 | 18.4 | 4.02 | 1222 | 7.0 | 6.54 | 13.0 | 82.7 | 2.1 | 2.0 | 0.2 |
| **B12** | 8.26 | 13.8 | 45.0 | 54.5 | 16.7 | 30.7 | 28.4 | 19.9 | 4.08 | 1160 | 7.2 | 5.17 | 12.0 | 82.8 | 1.9 | 3.3 | 0.0 |
| **C4** | 9.10 | 14.2 | 47.3 | 52.0 | 15.6 | 30.0 | 26.2 | 21.1 | 3.58 | 778 | 6.9 | 5.14 | 11.7 | 84.2 | 1.4 | 2.3 | 0.4 |
| **C8** | 7.72 | 11.9 | 39.9 | 51.7 | 15.4 | 29.8 | 26.2 | 18.8 | 3.16 | 943 | 6.9 | 5.81 | 16.8 | 79.9 | 1.0 | 2.1 | 0.2 |
| **C14** | 8.34 | 13.3 | 44.0 | 52.8 | 15.9 | 30.2 | 27.3 | 20.7 | 2.82 | 51 | 9.5 | 3.27 | 18.7 | 75.2 | 3.7 | 2.1 | 0.3 |
| **D16** | 7.49 | 12.0 | 39.1 | 52.2 | 16.0 | 30.7 | 25.3 | 17.2 | 2.42 | 498 | 6.5 | 4.41 | 7.9 | 89.6 | 0.7 | 1.6 | 0.2 |
| **D21** | 8.15 | 12.5 | 41.9 | 51.4 | 15.3 | 29.8 | 27.2 | 20.9 | 0.62 | 77 | 7.8 | 6.52 | 14.1 | 78.8 | 4.0 | 2.9 | 0.2 |
| **D25** | 8.17 | 12.5 | 41.3 | 50.6 | 15.3 | 30.3 | 25.5 | 18.7 | 3.55 | 978 | 6.9 | 5.30 | 14.0 | 81.1 | 3.2 | 1.5 | 0.2 |

**Appendix Table S6:**

**Serum analysis of a multiple-dose toxicity study.**

Mice (n = 3) were intranasal administrated with 2, 10 or 50 mg/kg of C6G25S once daily for 14 days and serum was collected for analysis. The vehicle control group were labeled as A2, A3, A11, 2 mg/kg-treated group as B1, B9, B12, 10 mg/kg-treated group as C4, C8, C14, and 50 mg/kg-treated group as D16, D21, D25. AST, aspartate aminotransferase; ALT, alanine aminotransferase; BUN, blood urea nitrogen; CREA, creatinine. Index: H= Hemolysis; L= Lipemia; F= Fibrin, N/A= Not observed abnormal. CREA: The linear range is 0.2~25 mg/dL, less than the linear range is presented as <0.20 mg/dL.

| **Treatment** | **ID** | **AST**  **(U/L)** | **ALT**  **(U/L)** | **BUN**  **(mg/dL)** | **CREA**  **(mg/dL)** |
| --- | --- | --- | --- | --- | --- |
| **Control** | **A2** | **168** | **50** | **23.1** | **0.33** |
|  | **A3** | **244** | **55** | **23.4** | **0.23** |
|  | **A11** | **75** | **27** | **22.2** | **<0.20** |
| **2 mg/kg** | **B1** | **92** | **31** | **25.1** | **0.25** |
|  | **B9** | **83** | **34** | **30.4** | **0.22** |
|  | **B12** | **52** | **31** | **22.9** | **<0.20** |
| **10 mg/kg** | **C4** | **38** | **26** | **25.3** | **<0.20** |
|  | **C8** | **118** | **35** | **19.4** | **0.22** |
|  | **C14** | **69** | **28** | **29.7** | **<0.20** |
| **50 mg/kg** | **D16** | **50** | **29** | **21.7** | **0.22** |
|  | **D21** | **220** | **38** | **21.3** | **<0.20** |
|  | **D25** | **43** | **24** | **21.6** | **<0.20** |

**Appendix Table S7:**

**RT-qPCR primers for the quantification of cytokine mRNA**

| **Genes** | **Forward primer (5’-3’)** | **Reverse primer (5’-3’)** |
| --- | --- | --- |
| **IL6** | **TACCACTTCACAAGTCGGAGGC** | **CTGCAAGTGCATCATCGTTGTTC** |
| **TNFα** | **GGTGCCTATGTCTCAGCCTCTT** | **GCCATAGAACTGATGAGAGGGAG** |
| **IFNγ** | **CGGCACAGTCATTGAAAGCCTA** | **GTTGCTGATGGCCTGATTGTC** |
| **IFNα** | **GGATGTGACCTTCCTCAGACTC** | **ACCTTCTCCTGCGGGAATCCAA** |
| **GAPDH** | **CGACTTCAACAGCAACTCCCACTCTTCC** | **TGGGTGGTCCAGGGTTTCTTACTCCTT** |
