# Supplementary material for: A siRNA targets and inhibits a broad range of SARS‐CoV‐2 infections including Delta variant
Source: EMBO Mol Med. 2022 Feb 21;14(4):e15298. doi: 10.15252/emmm.202115298 (PMC8988202; doi:10.15252/emmm.202115298)
Supplement: Supplementary file 3 — Expanded View Figures PDF [file EMMM-14-e15298-s006.pdf]

Expanded View Figures

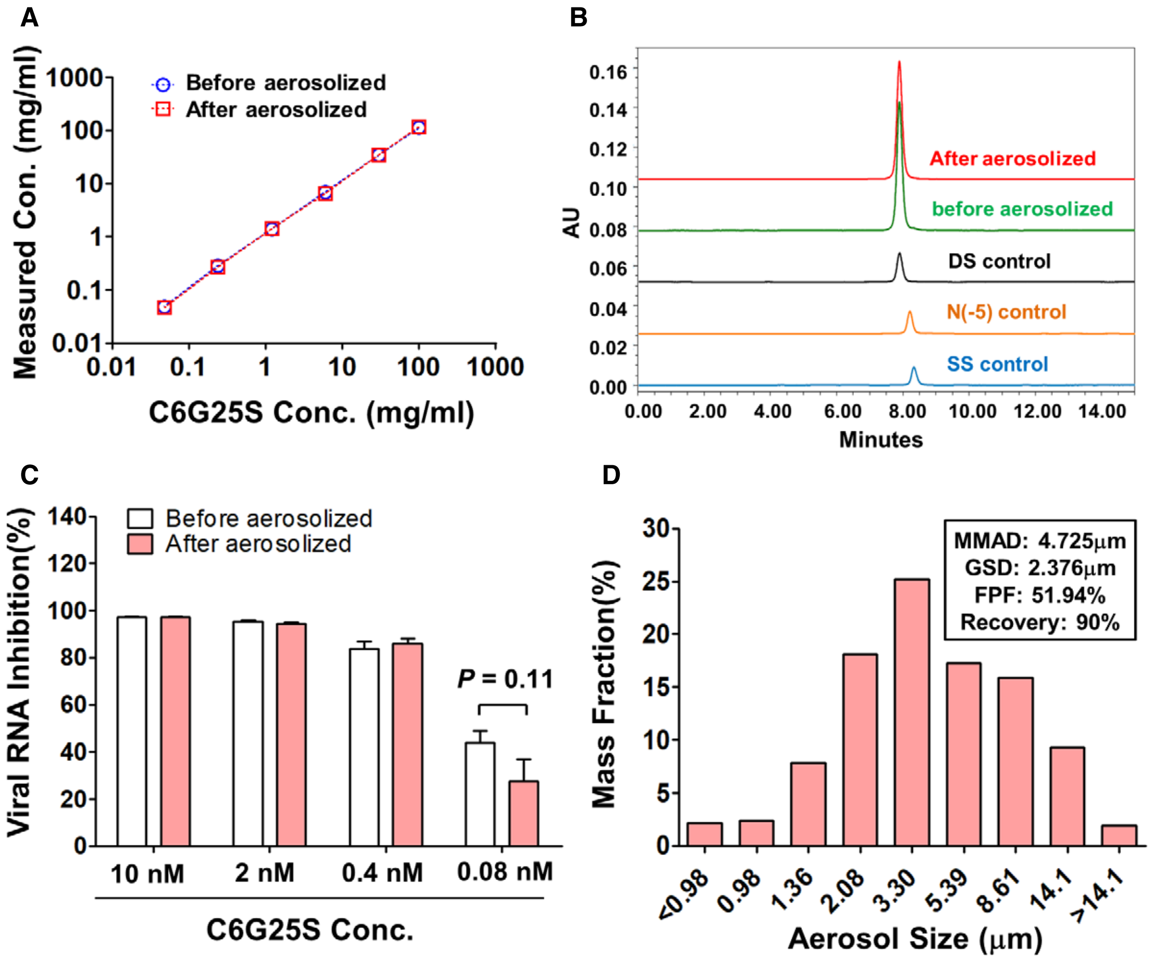

Figure EV1. Characterization and effectiveness of C6G25S in inhalation aerosol.

A The concentration of C6G25S before and after nebulization. The siRNA aerosol generated from 1 ml saline containing 125, 25, 5, 1, and 0.2 mg/ml of C6G25S was collected. The concentration of C6G25S before and after nebulization was measure by OD260.

B The integrity of siRNA before and after nebulization was analyzed via HPLC. The siRNA aerosol generated from 1 ml saline containing 6 mg/ml C6G25S was collected and analyzed compared with that before nebulization. Neither denaturation nor degradation was found after nebulization. DS control: Stock of double strand C6G25S. SS control: Antisense strand of C6G25S. *N*(−5) control: Double strand C6G25S with five bases truncated in both strands.

C The effectiveness of C6G25S before and after nebulization was determined via the inhibition of viral RNA. The siRNA aerosol was generated from 0.5 ml saline containing C6G25S 0.5 mg/ml. The RNA concentration was then determined by OD260 before nebulization and after siRNA aerosol was collected. Vero E6 cells were transfected with C6G25S (before or after nebulization) at different concentration for 24 h before infection with SARS-CoV-2 at a multiplicity of infection (MOI) of 0.1. The control cells were transfected with negative siRNA. The expression of viral envelope gene in the virus-infected cells was quantitated by RT-qPCR 24 h after infection. Data are presented as mean ± SD of three biological replicates. *P*-value by Student's *t*-test.

D Particle size distribution of nebulized siRNA aerosol generated from Aeroneb nebulizer and 2 ml normal saline containing 6 mg/ml C6G25S siRNA was analyzed using the next generation impactor (NGI). The experiment was performed in a laboratory accredited under ISO17025.

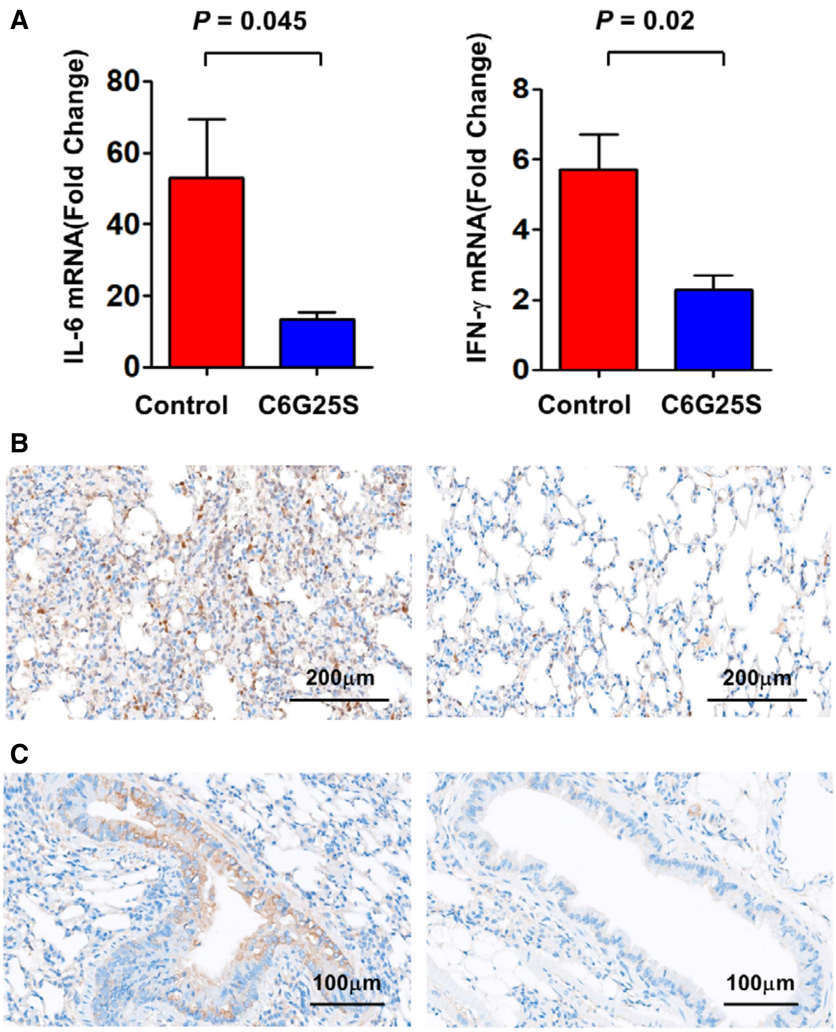

Figure EV2. C6G25S reduced the expression of inflammatory cytokines induced by SARS-CoV-2.

A K18-hACE2 transgenic mice ($n = 4$ per group) were challenged intranasally with 10^4 PFU of virus and cotreatment with 1.48 mg/l of C6G25S or vehicle control (saline) by AI for 30 min on day 0 (right after infection) and day 1. Mice were sacrificed at day 2 postinfection. Lung tissues of each mouse were collected, and RNA was extracted. The expression of IL-6 and IFN-γ were quantitated by RT-PCR. The amount of each cytokine relative to an uninfected sample is presented as mean ± SD. *P*-value by Student's *t*-test.

B Images of IHC staining of anti-IL6 (Brown color) in lungs of vehicle control (left) and C6G25S-treated group (right).

C Images of IHC staining of anti-IFN-γ (Brown color) in lungs of vehicle control (left) and C6G25S-treated group (right).

 © 2022 The Authors

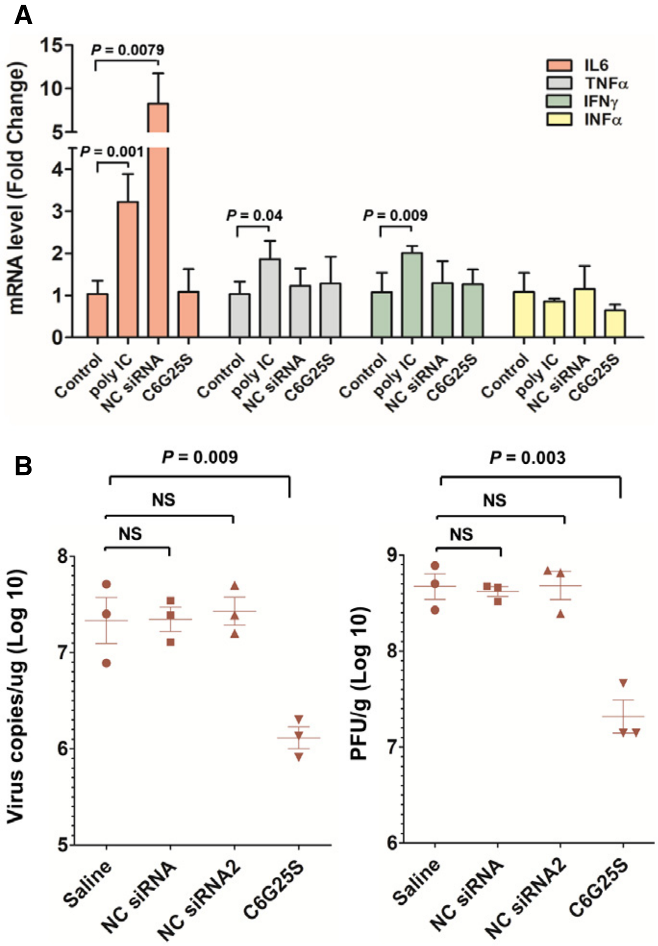

Figure EV3. C6G25S does not activate nonspecific immune response against SARS-CoV-2.

A ICR mice ($n = 3$) were treated with vehicle alone (saline), 2.5 mg/kg of poly(I:C) and negative control siRNA (an unmodified siRNA sequence used in the cell-based virus inhibition assay) via high volume (50 μl) of intranasal administration. Lung tissue was collected at 48 h after treatment. The mRNA expression of IL-6, TNF-α, IFN-α, and IFN-γ was quantitated by RT-PCR.

B hACE2 transgenic mice ($n = 3$) were pretreated with vehicle alone, 2.5 mg/kg of NC siRNA, NC siRNA 2, and C6G25S via high volume (50 μl) of intranasal administration at 48 h before virus infection. Viral RNA (left) and infectious virions (right) in lungs were quantitated with RT-qPCR and plaque forming assay, respectively, at 2 days postinfection.

Data information: Data are presented as mean ± SD. *P*-value by Student's *t*-test.

© 2022 The Authors

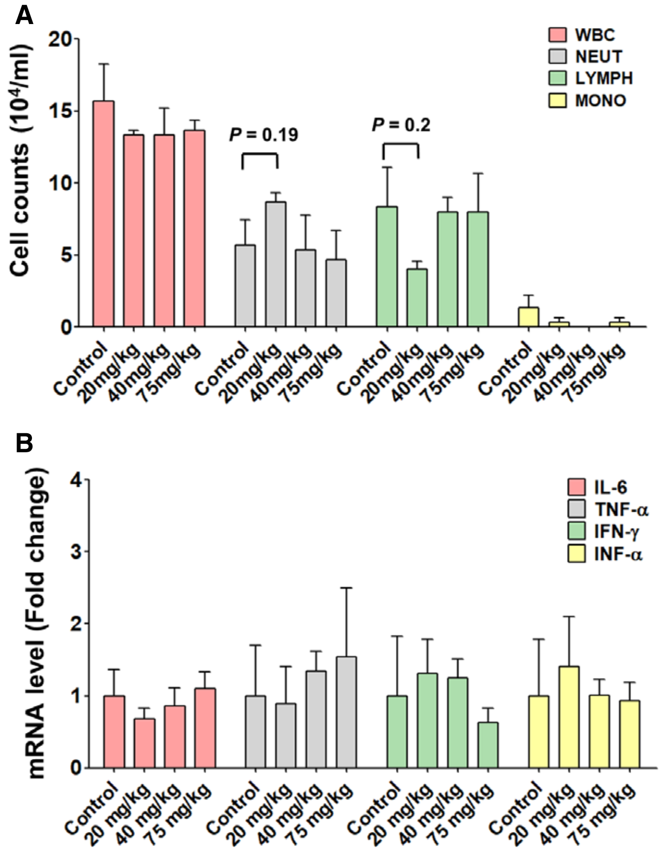

Figure EV4. No significant immune stimulation was observed in mice treated with high-dose of C6G25S.

A ICR mice (n = 3) treated with saline control or different dosages (20, 40, 75 mg/kg) of C6G25S by intranasal instillation. The bronchoalveolar lavage (BAL) was collected at day 2 after treatment. Different WBC cell counts were analyzed by a hematology analyzer. The cell counts are expressed as mean ± SD. All of the different treatment groups are statistically insignificant compared with the control in Student's *t*-test.

B Lung tissues of each mouse were collected, and RNA was extracted. The mRNA expression of IL-6, TNF-α, IFN-α, and IFN-γ was quantitated by RT-PCR. The relative amount of each cytokine is presented as mean ± SD. All of the different treatment groups are statistically insignificant compared with the control in Student's *t*-test.

 © 2022 The Authors
